# Photo-regulated disulfide crosslinking: a versatile approach to construct mucus-inspired hydrogels†

**DOI:** 10.1039/d4sc08284b

**Published:** 2025-02-25

**Authors:** Rui Chen, Krishnendu Das, Jun Feng, Boonya Thongrom, Rainer Haag

**Affiliations:** a Institut für Chemie und Biochemie, Freie Universität Berlin Takustraße 3 14195 Berlin Germany ruichen@zedat.fu-berlin.de haag@zedat.fu-berlin.de; b Organisch-Chemisches Institut, University of Münster Corrensstraße 40 48149 Münster Germany

## Abstract

The remarkable defensive ability of native mucus against pathogens has encouraged scientists to map its structure–-property correlation and its influence on immune defense mechanisms. However, its poorly defined structure, source-dependent composition, and low availability limit the usefulness of native mucus in the laboratory. This gap creates a strong demand for the development of synthetic mucus-mimetic materials. Here, we report a straightforward strategy for constructing mucus-mimetic hydrogels through photo-regulated disulfide crosslinking. Light-responsive 1,2-dithiolane attached to a linear polyglycerol sulfate (lPGS) backbone allows the macromolecular building blocks to crosslink and form the hydrogel, which mirrors the chemistry of native mucus hydrogel formation with its disulfide-linked mucin chains. The viscoelastic properties of the hydrogel can be easily tuned by controlling both the light exposure time and the number of 1,2-dithiolane units within the polymer backbone. Furthermore, localized UV irradiation allows for spatially resolved hydrogel formation. Importantly, this synthetic polymer can directly crosslink with native mucin, bovine submaxillary mucin (BSM), to convert it into a hydrogel at physiological pH. The versatility of this approach – hydrogel formation *via* photo-regulated disulfide crosslinking – can be used to develop a synthetic mucus model.

## Abstract 2

We report a photo-regulated disulfide crosslinking strategy to construct mucus-mimetic hydrogels. Light-responsive 1,2-dithiolane on linear polyglycerol sulfate (lPGS) enables dynamic crosslinking and forms a biohybrid hydrogel with native mucin.

## Graphical abstract

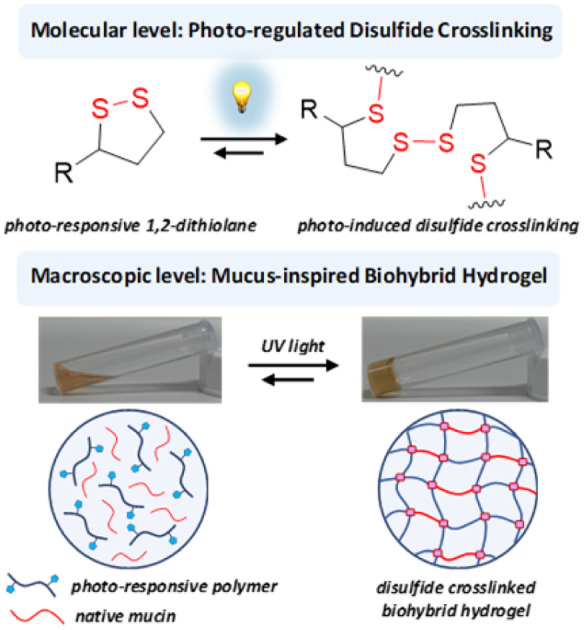

## Introduction

**Scheme 1:**
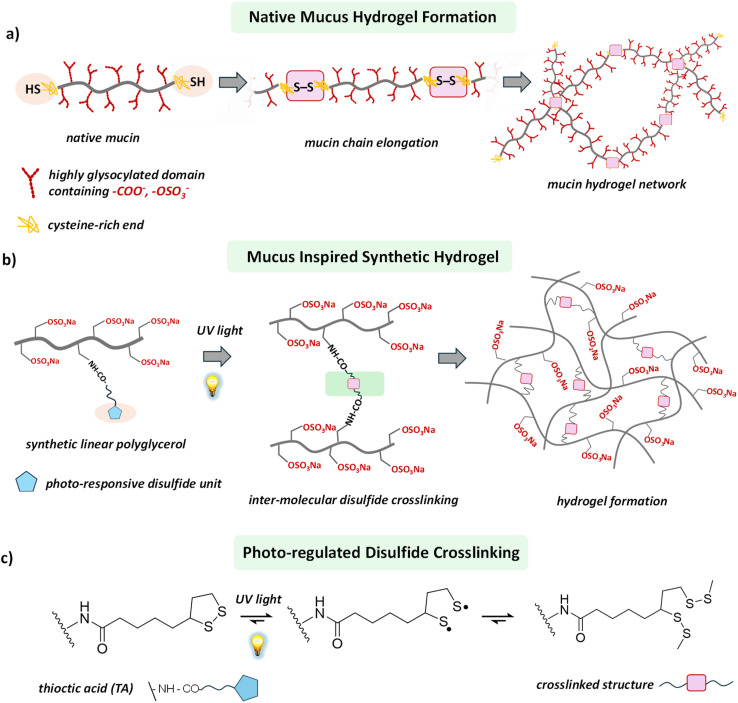
(a) Schematic illustration of mucus hydrogel formation through disulfide bond formation between the mucin proteins. (b) Representative structural illustration of our designed polymer structure which undergoes light regulated disulfide crosslinking between the macromolecular building blocks to form a hydrogel. (c) Light-regulated disulfide crosslinking chemistry of thioctic acid (TA).

Hydrogels play an important role in biomedical engineering, with their high water content, pliant elasticity, and allowing facile diffusion of bio-macromolecules.^1–3^ Consequently, hydrogels have found diverse applications in drug delivery, biosensors, tissue engineering scaffolds, and regenerative medicine.^4–10^ Synthetic hydrogels have served as models for biological viscoelastic materials such as the extracellular matrix and the mucus layer, helping researchers to understand these substances' roles and working principles in biology.^11–13^ Mucus is a crucial biological soft material – and an attractive research target – as it presents as a dynamic viscoelastic hydrogel, offering resistance to pathogens, particles, and toxic chemicals to protect epithelial cells from environmental injury.^12,14–17^ The viscoelastic properties of mucus originate from its component mucins, in particular the entangled fibrillar network that they form.^12,13,18–20^ Gel-forming mucin proteins contain cysteine-rich end groups that allow them to link together through disulfide bonds and thereby attain their mesh-like crosslinked structure (Scheme 1a).^18,21^ The mucus layer's remarkable ability to protect against pathogens has sparked great interest in understanding its underlying defensive mechanism.^12,22–24^ However, extracting native mucus in sufficient quantity and purifying its mucin for research is a complex task.^12^ The poorly defined structures and source-dependent composition of mucus also restrict its research.^12,18^

Many efforts have been made to design and develop mucus mimics,^12,14^ but the resulting materials fail to attain the inherent complexity of their biological counterpart, leaving them materially and functionally distant from native mucus. More importantly, most of the synthetic hydrogels are typically characterized by a static and non-dynamic network structure, unable to offer the dynamic viscoelastic properties relevant for developing artificial mucus.^25^ In the native mucus hydrogel, the viscoelasticity is controlled primarily by the manipulation of disulfide linkages between mucin proteins.^12,13,15,26^ Therefore, a synthetic building block whose viscoelastic behavior in the gel state can be tuned through controlled intermolecular disulfide bond formation would pave a broader path for developing mucus-mimetic hydrogels.

Recently, dynamic covalent chemistry has emerged as a powerful tool for constructing dynamic and adaptive hydrogels.^27–29^ Among its many approaches, thiol-to-disulfide chemistry has stood out because of its ubiquity in biological systems, the ease of breaking the disulfide bonds under reducing conditions, and the possibility of thiol exchange in a competitive environment.^3,30,31^ Throughout nature, intra- and intermolecular disulfide bridging defines the structure and functions of biopolymers.^32,33^ In the mucus hydrogel, mucin proteins contain cysteine-rich terminal domains that allow them to form elongated mucin chains through inter-mucin disulfide bond formation.^15,21^ This process generates a high-molecular-weight network structure that contributes significantly to the viscoelasticity of the mucus hydrogel (Scheme 1a).^16,17^ In a typical synthetic setup, an oxidizing agent converts the thiol groups, structurally encoded within the synthetic building blocks, to intermolecular disulfide bonds, thereby helping to form crosslinked networks and subsequently hydrogel.^3,26,30,31^ However, oxidizing agents and the required photo-initiators are often hazardous for biological studies, restricting these synthetic materials from biological evaluation. Moreover, control of the hydrogel's material properties over the spatial domain is a daunting task, as it requires local programming of the thiol-to-disulfide chemistry.

**Fig. 1:**
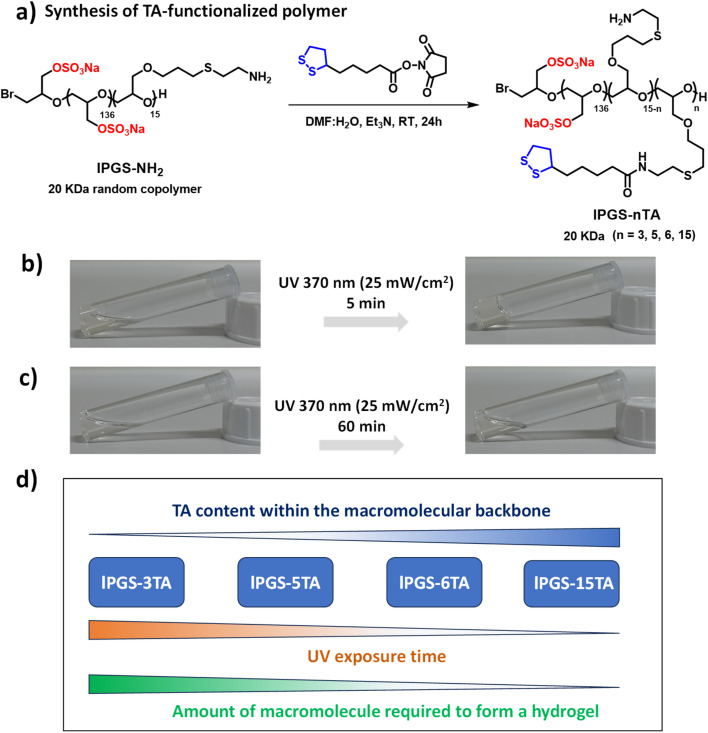
(a) Synthetic scheme of integrating thioctic acid (TA) within the linear polyglycerol sulfate (lPGS). (b) Vial images of lPGS-15TA (5% w/v) before and after 5 min UV (370 nm, 25 mW cm^−2^) irradiation in pH 7.4 PBS buffer at 25 °C. (c) Vial images of lPGS-3TA (10% w/v) before and after 60 min UV (370 nm, 25 mW cm^−2^) irradiation in pH 7.4 PBS buffer at 25 °C. (d) Correlation between the UV exposure time, TA contents within the polymer building blocks and amount of building block required to form the hydrogel.

Herein, we report a versatile approach to engineer a synthetic hydrogel, using a photo-regulated disulfide crosslinking strategy to develop a mucus-mimetic model hydrogel (Scheme 1b). Introducing light as a control tool offers the advantage of avoiding hazardous oxidizing agents and requirement of any photosensitizers.^34^ More importantly, the use of light as a trigger generates the scope of tuning the material properties of the hydrogel over the spatial domain as it can be delivered locally.^34–36^ We synthesized a linear polyglycerol sulfate (lPGS)-based macromolecular building block functionalized with light-responsive 1,2-dithiolane, which produces a hydrogel upon ultraviolet (UV) light irradiation. Structurally integrated light-responsive 1,2-dithiolane units within the lPGS building block allow it to commence inter-strand crosslinking through disulfide bond formation upon UV irradiation (Scheme 1c and Fig. 1a). Consequently, a giant crosslinked network is formed, which eventually turns into a hydrogel. We found that the viscoelasticity of the hydrogel can be tuned by varying the light exposure time as well as the number of 1,2-dithiolane units present within each macromolecular building block. These two parameters allow us to manipulate molecular-level crosslinking, ultimately translating into a tool for controlling macroscopic viscoelasticity.

## Results and discussion

Our primary objective was to develop a straightforward approach to constructing a mucus-inspired hydrogel through photo-regulated disulfide crosslinking between macromolecular building blocks. In order to develop the light-regulated hydrogels, we focused on 1,2-dithiolanes, a family of disulfide compounds with a highly strained five-membered ring.^37–39^ This family has gained enormous attention, as the 1,2-dithiolane group can undergo intermolecular crosslinking upon UV irradiation without photo-initiators (Scheme 1c).^34,40–42^ Consequently, it provides an alternative route to developing artificial disulfide-based biomaterials, where toxic and otherwise hazardous chemicals can be avoided. The motivations for choosing linear polyglycerol sulfate (lPGS) as the macromolecular building block are its ease of synthesis, multifunctionality, low intrinsic viscosity, and superior biocompatibility.^43–47^ Importantly, lPGS shares specific characteristics with native mucus, such as a polyglycerol backbone functionalized with amine groups providing a multivalent binding motif (Fig. 1a). The sulfate groups, which are also structural parts of native mucin, provide antiviral activity against various virus families.^14,17,44,45,47^

First, we synthesized a linear polyglycerol backbone by anionic ring-opening polymerization of ethoxyethyl glycidyl ether and allyl glycidyl ether, followed by deprotection in slightly acidic media.^43^ Next, the polymer was sulfated by following a reported protocol.^46^ The allyl glycidyl units on the backbone were then functionalized with cysteamine through thiol–ene chemistry and characterized by NMR (Scheme S1 and Fig. S1–S3†).^43^ Then, thioctic acid (TA), a biologically occurring small molecule that contains 1,2-dithiolane, was coupled with the polyglycerol-based backbone in a controlled way by varying the ratio between the polymer lPGS-NH_2_ and TA-NHS ester (Fig. 1a and S4†).^37^ A series of lPGS-*n*TA polymers (*n* = 3–15) were synthesized by linking different numbers of TA units within the backbone in a controlled way, as shown in Fig. 1a. All the lPGS-*n*TA polymers were soluble in aqueous buffer (pH 7.4, 10 mM PBS buffer) but could not form a hydrogel (Table S1, ESI†). Interestingly, upon UV light irradiation the synthesized polymers showed different gelation behavior. A 5% (w/v) free flowing solution of lPGS-15TA in PBS buffer, upon UV light irradiation for 5 min, formed a free-standing hydrogel (Fig. 1b), whereas a 10% (w/v) solution of lPGS-3TA could not form a hydrogel even after 60 min of UV light exposure (Fig. 1c).

Noticeably, the structural difference between these two polymers is limited to the TA components of the macromolecular backbone. We anticipate that, under UV exposure, TA units of the lPGS-15TA polymer start to crosslink intermolecularly to create a giant network structure, which in turn eventually immobilizes the solvent to create a self-supporting hydrogel. To verify this hypothesis, we performed time-resolved NMR studies of lPGS-15TA under UV exposure at two different concentrations (Fig. S5a and b†). The merging of the clearly resolved characteristic peaks from TA upon UV light irradiation, as indicated by yellow and blue coloration in Fig. S5a and b,† demonstrates the five-membered ring opening and polymerization. Meanwhile, we noticed that for both concentrations, the NMR kinetics (Fig. S5a and b†) showed the same trend, which means that the light-regulated crosslinking occurred in both cases. The difference in the macroscopic outcome is presumably because 3% (w/v) lPGS-15TA developed an insufficient crosslinked network and consequently remained in solution. By contrast, 6% lPGS-15TA formed a sufficiently extensive, crosslinked network, resulting in a free-standing hydrogel. This hypothesis was further supported by observing the case of lPGS-3TA, which, despite forming disulfide crosslinking as evident from the NMR spectrum (Fig. S5c†), could not form a hydrogel even at 10% (w/v) concentration (Fig. 1c). To further verify this hypothesis, we synthesized two more polymers with different TA contents and investigated their hydrogelation properties under UV exposure. We found that lPGS-5TA, at 10% (w/v) solution in PBS buffer, transformed into a free-standing hydrogel upon 10 min UV irradiation. Interestingly, lPGS-6TA, at 8% (w/v) solution in PBS buffer, formed a hydrogel after just 5 min of UV exposure (Table S1†). It is important to note that as the TA content of the backbone increases, both the UV exposure time and the amount of macromolecular building block required to form the hydrogel decrease (Fig. 1d and Table S1†).

In the next step, we employed oscillatory rheology to further investigate the hydrogelation behavior of lPGS-*n*TA and understand the significance of TA functionalization within the backbone. The mechanical properties of a hydrogel can be described by its viscoelastic properties, which can be estimated by oscillatory rheology experiments.^13,26^ Bulk rheological properties of the hydrogels were characterized using cone-and-plate geometry. A strain sweep test was carried out over the entire series to establish the linear viscoelastic region (LVE). We then performed a frequency sweep in the LVE region across the angular frequency (*ω*) range of 0.1–100 Hz to investigate the viscoelasticity of the hydrogels. In bulk rheological measurements, the storage modulus (*G*′) dominated over loss modulus (*G*′′), confirming that the intermolecular disulfide crosslinking of polymers induced hydrogelation.^13,26^

**Fig. 2:**
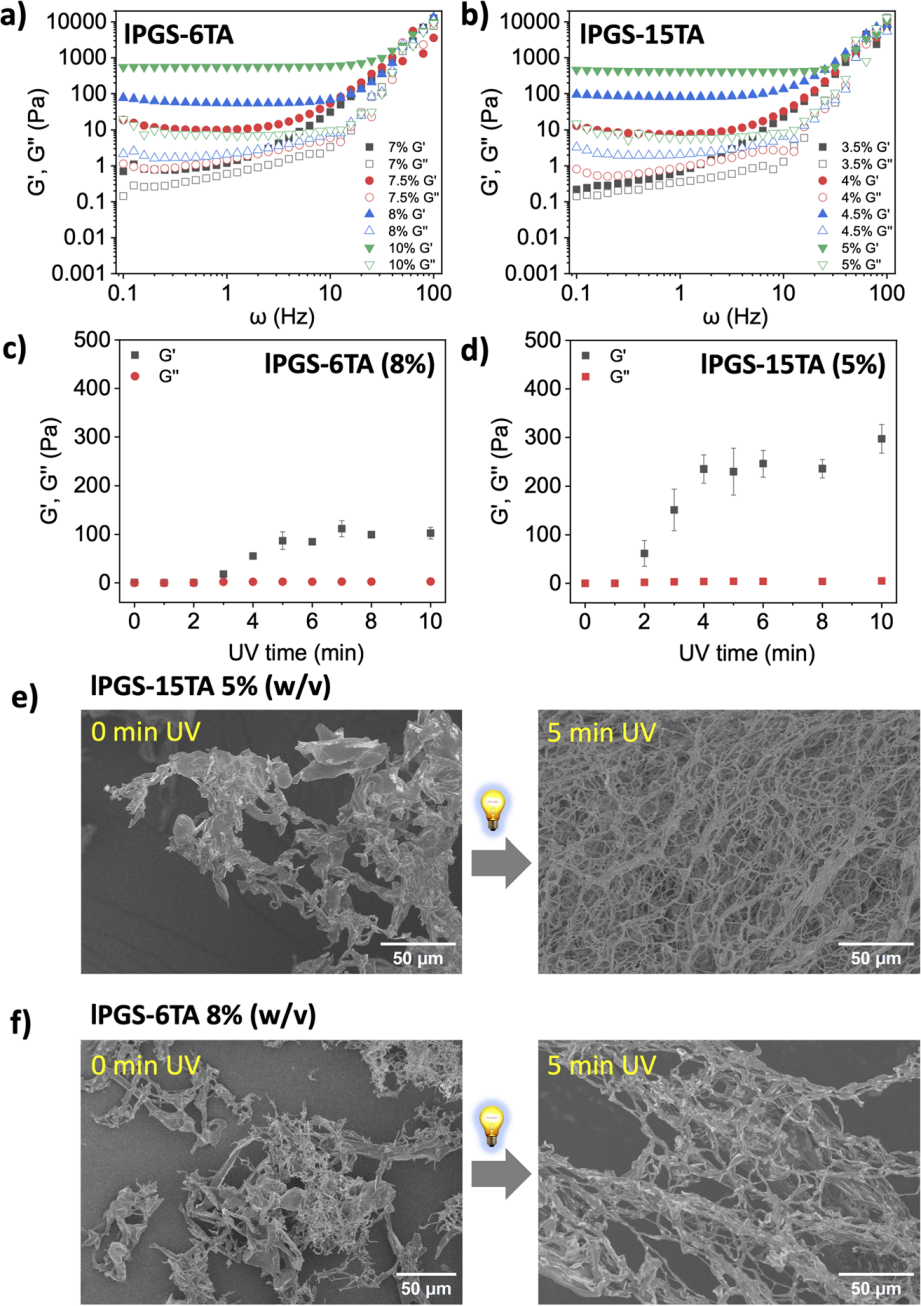
Storage (*G*′) and loss (*G*′′) moduli as a function of radial frequency *ω* for different concentrations of (a) lPGS-6TA and (b) lPGS-15TA. Changes in storage modulus (*G*′) and loss modulus (*G*′′) as a function of UV exposure time for (c) lPGS-6TA (8% w/v) and (d) lPGS-15TA (5% w/v). Samples were analysed at 1 Hz due to its physiological relevance.^49,50^ All the data were recorded at 1% fixed strain. (e) SEM images of lPGS-15TA (5% w/v) before and after 5 min UV irradiation. (f) SEM images of lPGS-6TA (8% w/v) before and after 5 min UV irradiation. UV irradiation conditions were fixed at 370 nm and 25 mW cm^−2^. All the samples for SEM were lyophilized polymer aqueous solutions or hydrogels that were prepared in PBS (pH 7.4) buffer at 25 °C. All the experiments were conducted in triplicate. The error bars have been omitted for clarity in (a) and (b).

In the first set of experiments, we investigated the critical gelation concentration (CGC)^48^ of lPGS-*n*TA under a fixed UV exposure time, or the minimum concentration required to form a hydrogel under a fixed UV exposure time. We fixed the UV exposure time at 5 min and the intensity at 25 mW cm^−2^, and we varied the concentration of lPGS-6TA during the frequency sweep at a constant strain of 1%, which is within the LVE. We found that at 7% (w/v) concentration of lPGS-6TA, the storage modulus (*G*′) and loss modulus (*G*′′) exhibited comparable behavior indicating solution state (Fig. 2a). Noticeably, at 7.5% (w/v) concentration of lPGS-6TA, *G*′ started to significantly dominate over the *G*′′, indicating gel-like behavior (Fig. 2a). Upon further increase in molecular concentration, the *G*′ kept dominating over *G*′′, indicating a value of around 7.5% (w/v) for the CGC of lPGS-6TA (Fig. 2a).

In the next experiment, we investigated the behavior of lPGS-15TA using the same experimental setup. Interestingly, here the CGC dropped to 4% (w/v): *G*′ began to dominate significantly over *G*′′ at 4% (w/v) concentration, then continued dominating with increase in molecular concentration (Fig. 2b). This change in the CGC of lPGS-TA under a fixed UV exposure time further reinforces the importance of TA count within the backbone. lPGS-15TA contains more TA units to form the crosslinked structure, and therefore the CGC is much lower than that of lPGS-6TA.

It is important to mention that, for both lPGS-15TA and lPGS-6TA, at CGC and under fixed UV irradiation, *G*′ values were observed in the range of 7–10 Pa, while *G*′′ remained around 1 Pa at 1 Hz. The native mucus hydrogel, which exhibits viscoelastic shear-thinning properties, shows *G*′ and *G*′′ within the range of 1–20 Pa.^12^ The observed values fall within the previously reported range for healthy human lung mucus (*G*′ = 1–10 Pa).^49–51^ It is also important to notice that in the frequency sweep rheology of native airway mucus, both *G*′ and *G*′′ exhibit a sharp upward turn above 10 Hz, while a constant plateau is observed at lower frequencies, which reflects the equilibrium viscoelastic nature of the mucus.^26^ A similar trend was observed in our developed material (Fig. 2a and b). Therefore, the hydrogels formed from lPGS-*n*TA, not only share structural similarities but also achieved mechanical properties comparable to those of native mucus.^13^

In the next step, we looked at the time scale of UV exposure to gain further insights into the role of light-regulated disulfide crosslinking in hydrogel formation. Towards this objective, we prepared a series of vials containing a fixed concentration of lPGS-*n*TA and irradiated them independently with UV light for different time spans before performing rheology measurements. We fixed the concentration of lPGS-6TA at 8% (w/v), which is close to its CGC, and varied the UV exposure time during the viscoelasticity measurement at a fixed radial frequency of 1 Hz. *G*′ did not change noticeably in the beginning, but after 3 min, *G*′ rapidly increased until becoming saturated at the 5 min mark (Fig. 2c and S6a†). Thereafter, the *G*′ did not change upon further UV exposure, indicating saturation in the light-regulated disulfide crosslinking. Interestingly, in the case of lPGS-15TA, at 5% (w/v), *G*′ began increasing within 2 min and became saturated after around 4 min of UV exposure (Fig. 2d and S6b†). More importantly, it is significant to note that, despite sharing the same molecular structure and differing only in their TA content, 5% (w/v) lPGS-15TA produced a much higher *G*′ value than 8% (w/v) lPGS-6TA. These two observations clearly reinforce the importance of intermolecular disulfide crosslinking under UV exposure. Increased TA unit count within the backbone enabled sufficient crosslinking after shorter UV exposure time spans and produced more crosslinking at longer timescales, translating eventually into a stronger hydrogel at lower macromolecular concentration. This conclusion is further supported by the observation that lPGS-15TA at 10% (w/v) concentration produced a much stronger hydrogel with significantly higher *G*′ values than the previous two cases using the same experimental setup (Fig. S7†).

This light-regulated intermolecular crosslinking and its impact on the rheological behavior of the system compelled us to investigate the structural properties of the hydrogels by using scanning electron microscopy (SEM). As we have already seen, lPGS-15TA cannot form a hydrogel at 5% (w/v) concentration without UV exposure; consequently, the corresponding SEM image shows only undefined structures (Fig. 2e left and S8a†). But upon UV exposure for 5 min, the free-flowing solution immediately transformed into a free-standing hydrogel with densely packed fibrillar structures (Fig. 2e right and S8b†). Similarly, lPGS-6TA at 8% (w/v) concentration in PBS buffer showed no prominent structure, whereas upon UV exposure, it transformed into a hydrogel, displaying a fibrillar structure as revealed by SEM (Fig. 2f, S8c and d†). It is important to note that, after UV exposure, lPGS-15TA produced a much more densely packed fibrillar network at lower molecular concentration than lPGS-6TA, a difference that was also reflected in the corresponding rheological outcome (Fig. 2c and d).

The data presented so far show that lPGS-*n*TA forms a hydrogel through light-regulated intermolecular disulfide crosslinking, which is mechanistically similar to mucus hydrogel formation through disulfide crosslinking between individual mucin proteins. Additionally, the mechanical strength of the hydrogels can be tuned by the controlled grafting of TA units within the macromolecular backbone and by adjusting the UV exposure time. Furthermore, these hydrogels exhibited a range of viscoelasticity comparable to that of healthy human sputum.

**Fig. 3:**
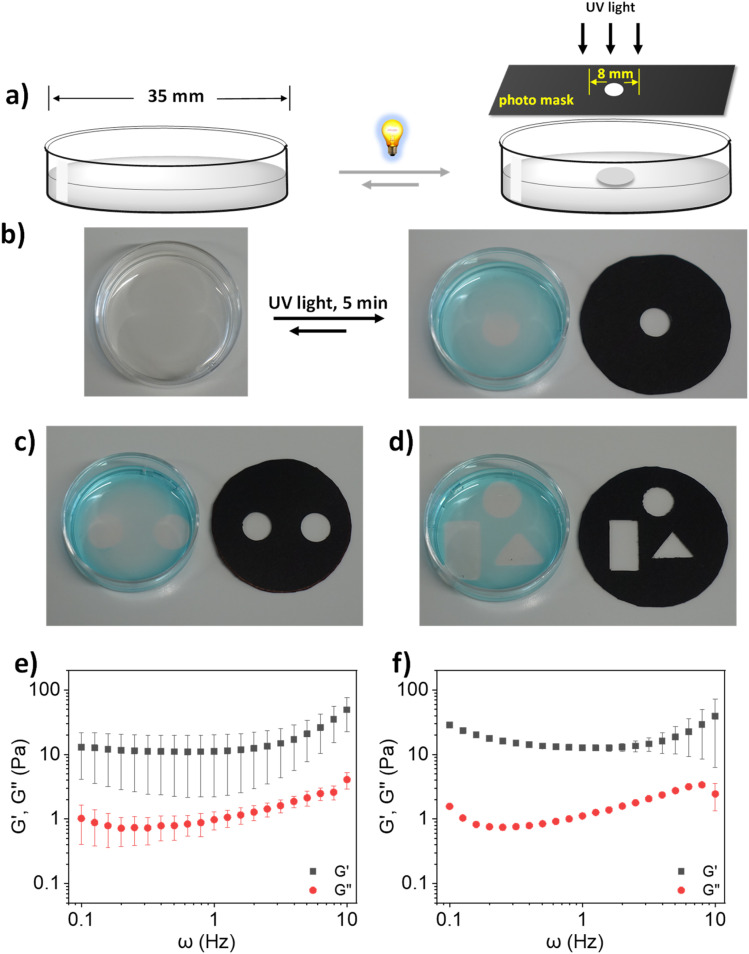
(a) Schematic illustration of light-regulated local gel formation using a photomask. (b) Light-regulated local gel formation of lPGS-15TA at CGC (4% w/v). (c) and (d) Regulating local gel formation by changing the geometry of the photomask. Storage (*G*′) and loss (*G*′′) moduli as a function of radial frequency *ω* for lPGS-15TA (4% w/v) in (e) spatially formed gel and (f) homogeneous gel. UV irradiation conditions were fixed at 370 nm and 25 mW cm^−2^. All experiments for (e) and (f) were conducted in triplicate.

At this point, we were intrigued by the possibility of spatially resolved hydrogel formation. Spatial heterogenicity is another crucial characteristic of mucus which plays important roles in defense against pathogen infection. The primary origin of this spatial heterogenicity is the difference in the density of inter-mucin disulfide bonds.^52^ It prompted us to investigate whether our developed model system can also permit the formation of spatial hydrogels which is directly correlated with the formation of enhanced disulfide crosslinking within a localized space than the other parts. A key advantage of light as a trigger is its spatial dimension, as it can be supplied locally.^36,53^ After seeing lPGS-15TA form a hydrogel upon UV irradiation, we were curious to explore whether local gel formation could be achieved by locally irradiating an lPGS-15TA solution using a photomask (Fig. 3a). Towards this objective, we prepared a 4% (w/v) lPGS-15TA polymer solution in a plastic Petri dish with a diameter of 35 mm (Fig. 3b). To apply the local UV irradiation, we covered the Petri dish with one black photomask with an 8 mm diameter hole in its center (Fig. 3a). After 5 min of UV irradiation through the photomask, a transparent gel “island” was indeed obtained at the center of the Petri dish, while the other areas remained in solution (Fig. 3b). After 5 min of UV irradiation, we added 10 μL of methylene blue (2 mM) to the Petri dish to better visualize the local gel formation. Methylene blue, a water-soluble dye, remained distributed throughout the water phase, while the center of the Petri dish, where the local gel formed, remained a transparent solid. Photos taken before and after the UV irradiation show clearly that local UV light delivery *via* a photomask can indeed cause spatial gel formation (Fig. 3b). To further verify this spatially resolved gel formation under local delivery of UV light, we repeatedly altered the photomask geometry and optical images corresponding to the circular, square or triangular gels shown in Fig. 3c and d clearly show localized gel formation in each case. We also scooped out the locally formed hydrogel to investigate its viscoelasticity. We found that its viscoelasticity was in the range of human healthy sputum at 1 Hz (*G*′ = 1–10 Pa), comparable to the homogeneously formed hydrogel^13,49,50^ (Fig. 3e and f).

**Fig. 4:**
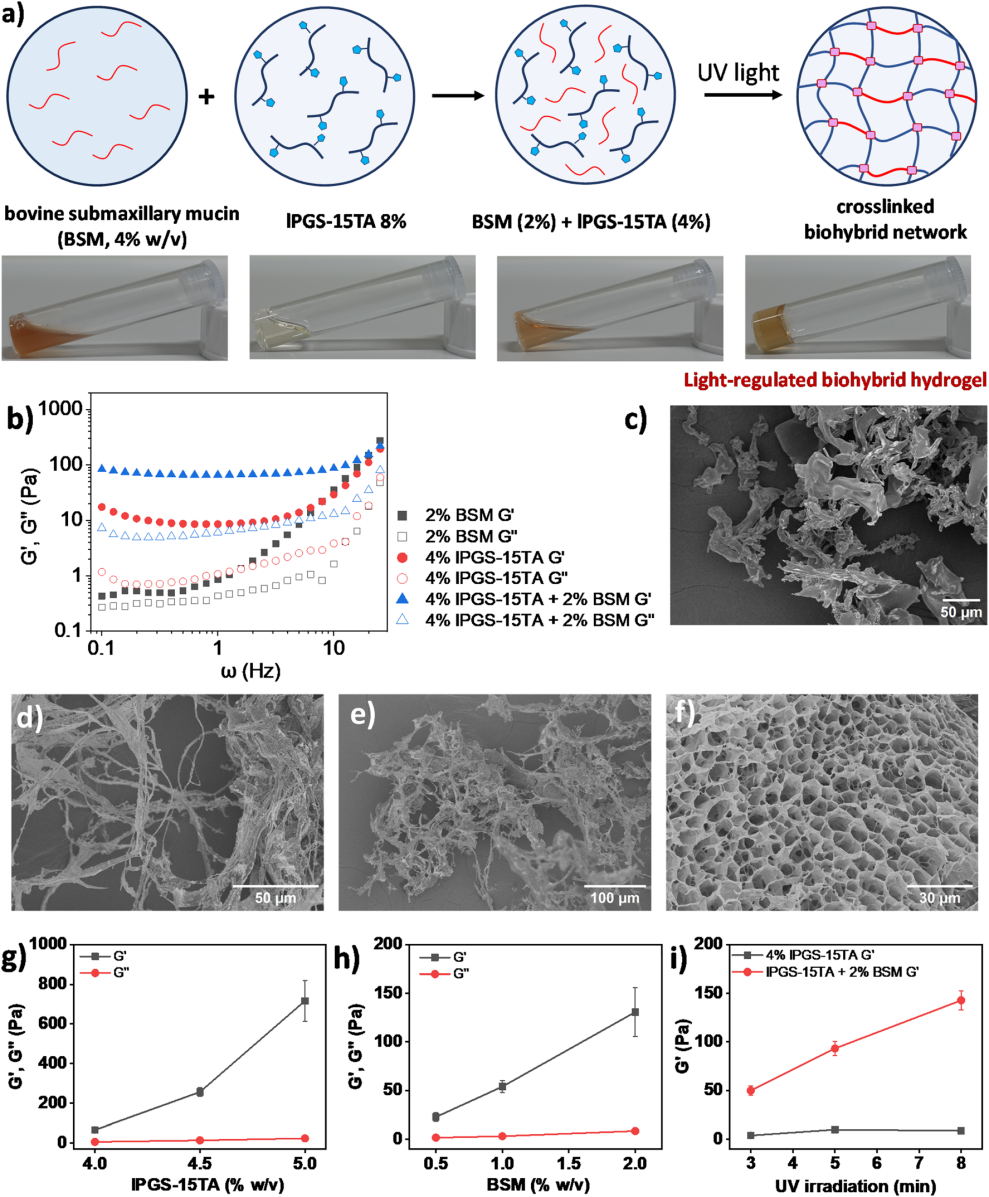
(a) Schematic presentation and vial images of light regulated biohybrid gel formation. (b) Storage (*G*′) and loss (*G*′′) moduli as a function of radial frequency *ω* for lPGS-15TA (4%), BSM (2%) and their mixture upon UV irradiation. (c) SEM image of lPGS-15TA (4% w/v) without UV irradiation. (d) SEM image of BSM (2% w/v) without UV irradiation. (e) SEM image of the mixture of lPGS-15TA (4% w/v) and BSM (2% w/v) without UV irradiation. (f) SEM image of the mixture of lPGS-15TA (4% w/v) and BSM (2% w/v) after UV irradiation. (g) At a fixed concentration of BSM (2% w/v), storage (*G*′) and loss (*G*′′) moduli of the biohybrid hydrogel change as a function of the lPGS-15TA concentration. (h) At a fixed concentration of lPGS-15TA (4% w/v), storage (*G*′) and loss (*G*′′) moduli of the biohybrid hydrogel change as a function of the BSM concentration. (i) At a fixed concentration of lPGS-15TA (4% w/v) and BSM (2% w/v), storage (*G*′) and loss (*G*′′) moduli of the biohybrid hydrogel change as a function of UV irradiation time. For a clear comparison, all *G*′/*G*′′ were taken from 1 Hz in experiments (g)–(i). The irradiation conditions were 5 minutes of 370 nm and 25 mW cm^−2^ UV light. All the experiments were conducted in triplicate. The error bars have been omitted for clarity in (b).

With these results in hand and in light of current challenges in mucus research, we were intrigued to investigate whether the synthesized polymer could directly crosslink with native mucin through light-regulated disulfide bond formation to create a biohybrid hydrogel, specifically one that could address certain shortcomings of today's standard mucus model. Biohybrid hydrogels, which consist of both natural and synthetic components, can leverage their combined advantages to provide superior three-dimensional (3D) model systems for biological research.^54^ In general, isolating native mucin in bulk and purifying it for experimental purposes is a complex task. Moreover, this sophisticated process often destroys the physicochemical structures of native mucins.^12,13,54^ Commercially available bovine submaxillary mucin (BSM), which is regarded as the model system for human mucus,^55^ does not form a hydrogel at physiological pH and requires high concentration to produce viscoelasticity comparable with native mucus; these traits reduce its utility as a model mucus system in laboratory setups.^54^ It has been shown that BSM only begins to exhibit viscoelasticity comparable to healthy sputum at a concentration range of 10–14% (w/v).^55^ We therefore attempted to reduce the required amount of BSM to form a hydrogel at physiological pH by using our synthesized polymer as an assistant molecule. Interestingly, we found that mixing BSM (2% w/v) with lPGS-15TA (4% w/v) formed a hybrid hydrogel with significant viscoelasticity upon UV exposure (Fig. 4a).

In the next step, we used bulk rheological measurement to gain further insights into this hybrid hydrogel formation (Fig. 4b). At 4% w/v, lPGS-15TA remained as a free-flowing solution, as reflected in bulk rheology measurements showing very low *G*′ and *G*′′ values during frequency sweep under a fixed strain of 1% (Fig. S9a†). The addition of BSM (2% w/v) within 4% w/v lPGS-15TA solution produced no significant change in viscoelasticity (Fig. S9a†). Noticeably, exposing this mixed solution to UV light for 5 min transformed the solution to a free-standing hydrogel. Rheology measurements also showed a significant increase in *G*′, indicating a gel-like hybrid material with considerably improved elasticity (Fig. 4b). It is important to note that *G*′ of the hybrid system after UV light exposure was much higher than *G*′ of the individual components. These two observations suggest the joint participation of both lPGS-15TA and BSM in the hybrid hydrogel formation, with lPGS-15TA helping BSM to create a crosslinked network structure. The light-regulated disulfide bond formation takes place with the thiol groups from BSM as well.^54^ We performed SEM analysis to gain structural information on the systems. Without UV irradiation, lPGS-15TA (4% w/v) presented as an undefined structure (Fig. 4c and S10a†), while 2% BSM presented random long fibrillar structures (Fig. 4d and S10b†), unsurprising for a high-molecular-weight protein. The mixture of lPGS-15TA (4% w/v) and BSM (2%) also showed irregular fibrillar structures (Fig. 4e and S10c†), while after 5 min under UV light, the mixture formed a 3D porous network structure as shown in Fig. 4f and S10d.† These SEM images provide direct evidence that lPGS-15TA helps BSM to form a crosslinked network structure, which eventually produces a porous hydrogel analogous to native mucus.^56^

In the next step, we systematically varied the concentrations of both components to better understand the biohybrid hydrogel. In the first set of experiments, we fixed the concentration of BSM at 2% (w/v) and varied the concentration of lPGS-15TA. We found that with increasing concentration of lPGS-15TA, *G*′ kept increasing during frequency sweep under a constant strain of 1% (Fig. 4g and S11†). Noticeably, *G*′ of the hybrid hydrogel always remained higher than *G*′ of lPGS-15TA alone (Fig. 4g and S11†). It is worth noting that without UV irradiation all samples presented as free-flowing solutions with low *G*′ and *G*′′ (Fig. S9b and c†). On the other hand, with the same experimental setup and with the lPGS-15TA concentration fixed at 4% (w/v), increasing the concentration of BSM led to a significant increase in *G*′ with negligible change in *G*′′ (Fig. 4h and S12†). This result indicates improvement in the material's elastic behavior at increased concentrations of BSM. To further verify the participation of BSM crosslinking with lPGS-15TA to form a hybrid hydrogel – as distinct from a crowding effect from BSM – we fixed the concentration of lPGS-15TA and BSM and varied the UV exposure time. We found that, with increased UV exposure time, *G*′ of lPGS-15TA alone changed only slightly, whereas *G*′ of the hybrid system increased significantly – a result that indicates direct participation of BSM in the hybrid hydrogel formation through light-regulated disulfide crosslinking (Fig. 4i and S13†). These experiments further establish that the light-responsive polymer lPGS-15TA can assist BSM to form a network structure and recreate the hydrogel state of native mucus. The viscoelasticity of the biohybrid hydrogels could be adjusted either by varying the component concentration or by simply varying the UV irradiation time (Fig. 4g–i).

Among the molecular structural features of mucin, three properties carry the strongest influence on the functional properties of mucus: inter-mucin disulfide bonds, extended glycosylic hydrophilic domains, and sulfate-bearing glycans.^12,18,21^ It is a challenging task for a single synthetic building block to capture all of these structural features along with functional macroscopic properties such as viscoelasticity. Moreover, a single synthetic building block that supports tunable viscoelasticity is also a key achievement, as it reduces the structural interference during biological studies. As a model system, the developed light-regulated hydrogel based on lPGS-*n*TA offers most of the key structural features of mucin, such as: an amine-functionalized polyglycerol backbone that provides multivalent binding motifs akin to mucin glycoproteins; sulfated units analogous to mucin; the intermolecular disulfide crosslinking that is a hallmark of mucus hydrogel formation;^13^ variable viscoelasticity against different disulfide crosslinking density; comparable viscoelasticity to human healthy sputum; and spatially resolved gel formation to capture the spatial heterogeneity of mucus hydrogel. Perhaps most importantly, the use of light sidesteps the potential hazards of using oxidizing agents in biological studies.

## Conclusion

In summary, we have designed and developed a versatile synthetic platform to construct mucus-inspired hydrogels. The macromolecular building blocks lPGS-*n*TA undergo intermolecular disulfide crosslinking through UV irradiation acting upon photo-responsive 1,2-dithiolane units. Importantly, intermolecular disulfide bond formation directly regulates the viscoelasticity of the material, just as with native mucus. The TA units can be easily integrated within the backbone by using simple NHS-ester activation chemistry and the number of TA units within the macromolecular backbone can be well controlled by regulating the ratio between the reagents. The straightforward, controlled integration of 1,2-dithiolane units into the macromolecular building blocks may prove useful for other macromolecular building blocks by choosing proper functionalities. Moreover, the photo-regulated crosslinking reaction requires no photo-initiators or oxidizing reagents, reducing the risk of chemical hazards during biological experiments. The viscoelasticity of the system can be regulated by tuning three parameters: TA content within the backbone, UV exposure time, and the concentration of the macromolecular building blocks. The importance of using light as a regulatory handle relies on the scope of providing it externally to regulate the material properties. Local UV light delivery *via* a photomask enables control of macroscopic properties across the spatial domain, as seen in the localized gel formation we demonstrated. The synthetic molecule lPGS-15TA can assist the native mucin BSM in forming a biohybrid hydrogel at physiological pH and concentration. The light-regulated disulfide chemistry at the molecular level offers exceptional versatility in tailoring the macroscopic properties of the material. This versatile approach of photo-regulated disulfide crosslinking strategy creates new opportunities, not only in developing artificial mucus but also in designing new biomimetic and biohybrid materials as the disulfide bond is ubiquitous in biological systems.

## Data availability

The data supporting this article have been included as part of the ESI.†

## Author contributions

R. C. designed the project, conducted all experiments, and wrote the manuscript. K. D. assisted with data analysis and manuscript preparation. J. F. supported polymer synthesis, and B. T. contributed to the rheology study. R. H. supervised the entire project. All authors reviewed and edited the manuscript.

## Conflicts of interest

There are no conflicts to declare.

## Supplementary Material

SC-016-D4SC08284B-s001
