# Supplementary material for: Photo-regulated disulfide crosslinking: a versatile approach to construct mucus-inspired hydrogels
Source: Chem Sci. 2025 Feb 25;16(13):5528–37. doi: 10.1039/d4sc08284b (PMC11851172; doi:10.1039/d4sc08284b)
Supplement: SC-016-D4SC08284B-s001 [file SC-016-D4SC08284B-s001.pdf]

Supplementary Information (SI) for Chemical Science.
This journal is © The Royal Society of Chemistry 2025

SUPPORTING INFORMATION

Photo-regulated Disulfide Crosslinking: A Versatile Approach to Construct Mucus-Inspired Hydrogels

Rui Chen*[a], Krishnendu Das[b], Jun Feng[a], Boonya Thongrom[a], Rainer Haag*[a]

[a]Institut für Chemie und Biochemie, Freie Universität Berlin, Takustraße 3, Berlin 14195, Germany.

[b]Organisch-Chemisches Institut, University of Münster, Corrensstraße 36, 48149 Münster, Germany.

Contents

Materials and Instrumentation 2
Polymer Synthesis and Characterisation 3
Synthesis of Poly (ethoxyethyl glycidyl ether)-Poly (allyl glycidyl ether) (PEEGE-PAGE) (lPG) 3
Synthesis of Sulfated PEEGE-PAGE (lPGS) 4
Synthesis of aminated lPGS (lPGS-NH_2) 5
Synthesis of lPGS-nTA 6
Time-resolved NMR 10
Gel preparation and Summary of gelation behaviour of lPGS-nTA 11
Oscillatory Rheology of lPGS-nTA hydrogels 12
Scanning Electron Microscope (SEM) images of lPGS-nTA 12
Oscillatory Rheology of mixture of lPGS-15TA and BSM 13
Scanning Electron Microscope (SEM) images of biohybrid hydrogels 14
Oscillatory Rheology of biohybrid hydrogels 14
References 15

Materials and Instrumentation

Materials

All the reagents and solvents were purchased from commercial suppliers and used without further purification. Benzoylated cellulose dialysis tubes (2 kDa, 32 mm width) and Bovine submaxillary mucin (**BSM,** LOT: 3829388) were purchased from Merck (Darmstadt, Germany).

Instrumentation

^{1}H NMR spectra were recorded in a Bruker AMX 500 MHz (Bruker Corporation) and the spectra were calibrated against TMS as the internal standard. Chemical shifts (δ) are reported in ppm via the deuterated solvent peak as the standard.

Weight average molecular weight (Mw) and polydispersity index (Đ) of the polymers were measured by size exclusion chromatography (SEC). Gel permeation chromatography (GPC) measurements in water were performed with an Agilent 1100 equipped with an automatic injector, isopump, and Agilent 1100 differential refractometer (Agilent Technologies, Santa Clara, CA, USA). The PSS Suprema (precolumn), 1× with pore size of 30 Å, 2× with pore size of 1000 Å (all of them with a particle size of 10 µm) column, was calibrated against Pullulan standards prior to measurements. In each measurement, 50.0 µL of a sample with a concentration of approximately 3.0 mg mL^{-1} was injected into the columns using the autosampler.

UV lamp PR160L-370nm Gen 2 was purchased from Kessil LED Lights for UV irradiation. The wavelength is 370 nm. 50% of the intensity (ca. 25 mW/cm^2) was applied in all the experiments. 5 cm distance was always maintained between the samples and the UV lamp.

Scanning electron microscope (SEM)

The morphologies of the hydrogels were investigated with a field emission scanning electron microscope (FE-SEM, Hitachi SU8030, Japan) at an accelerating voltage of 5 kV, a current of 10 µA and a working distance (WD) of around 8.3. The samples were dried under high vacuum and coated with a 5 nm gold layer by using a sputter coater (Emscope SC 500, Quorum Technologies, UK) for 20 s at 30 mA, 10-1 Torr (1.3 mbar) under argon atmosphere.

Oscillatory Rheology

The mechanical properties of a hydrogels can be described by its viscoelastic properties. They were estimated by oscillatory rheology experiments. It was measured and characterized by the Kinexus rheometer (NETZSCH GmbH, Selb, Germany). A cone and plate geometry, 20 mm in diameter was used for these measurements, with the average normal force maintained at ≈0.1 N at 25 °C. The data were

analysed by an oscillatory frequency sweep strain-controlled test with 1% strain (which is obtained from a linear viscoelastic range of an amplitude sweep test and frequency 0.1-100 Hz).

Polymer Synthesis and Characterisation

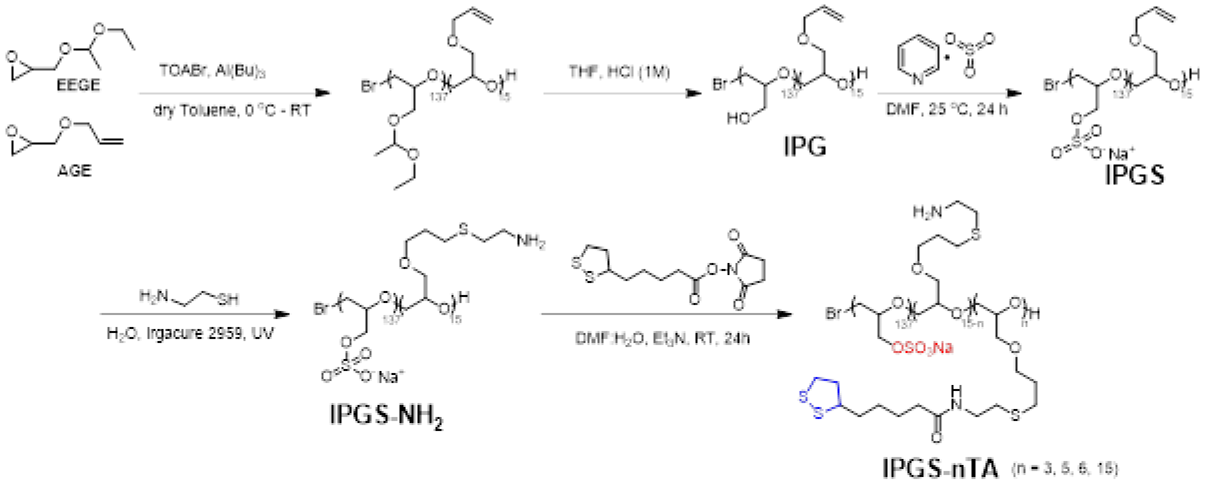

Scheme S1. Synthesis of **lPGS-nTA**.

Synthesis of Poly (ethoxyethyl glycidyl ether)-Poly (allyl glycidyl ether) (PEEGE-PAGE) (lPG)

The random copolymer linear polyglycerol backbone **lPG** was synthesized by following the reported protocol.[1] **lPG** (~ 10 KDa) with 15 allyl groups was targeted via ring-opening anionic polymerization of acetal-protected glycidyl monomers (EEGE) and allyl glycidyl ether (AGE) with the initiator of tetraoctylammonium bromide, followed by acetal deprotection under acidic conditions in THF.

Firstly, in a 100 mL round Schlenk flask tetraoctylammonium bromide (TOABr) (570 mg, 1 mmol, 1.05 eqv.) was heated to melting temperature (115°C oil bath) under vacuum to remove the trace water for 2 hours. Under nitrogen atmosphere, 50 mL dry toluene was added into the Schlenk flask to dissolve the initiator. After cooling down and the flask was set in ice bath, then 20 mL EEGE (19.74g, 135 mmol, 137 eqv.) and 1.75 mL AGE (1.687g, 15 mmol, 15 eqv.) were added into the solution. The polymerization was started by adding the 2.73 mL triisobutylaluminium (3 mmol, 3 eq.) at a constant temperature of 0 °C. The reaction mixture was allowed to warm up to room temperature and stirred overnight. After polymerization, 1 mL methanol was added to stop the reaction. A colorless polymer was obtained after evaporation of toluene under reduced pressure. Then the product was dissolved into diethyl ether and centrifuged to remove the residues of initiator and catalyst. To remove the acetal-protection from PEEGE, after evaporation of diethyl ether, the compound was dissolved into THF (120 mL) and an appropriate amount of hydrochloric acid aqueous solution (37%) was added. The precipitation appeared immediately under acidic condition. The mixture was stirred for 2 hours at room temperature. Subsequently, the supernatant was decanted, and the polymer was washed with THF, and then further purified by dialysis in methanol.

The ratio of EEGE/AGE was calculated from ^{1}H-nuclear magnetic resonance (^{1}H-NMR) spectra. The molecular weight was measured by gel permeation chromatography (GPC). [1b]

^{1}H-NMR (500 MHz, MeOD, 300 K) of **lPG**: Repeating units δ = 3.5-4.1 (polymer backbone: C***H***$_2$-C***H***(C***H***$_2$O)-O, O-C***H***2-CH=CH$_2$), 5.15-5.33 (C***H***$_2$=CH-, 2H), 5.9-6.0(CH$_2$=C***H***-, 1H). M_w, GPC, H_2O = 12503, M_w/M_n = 1.4.

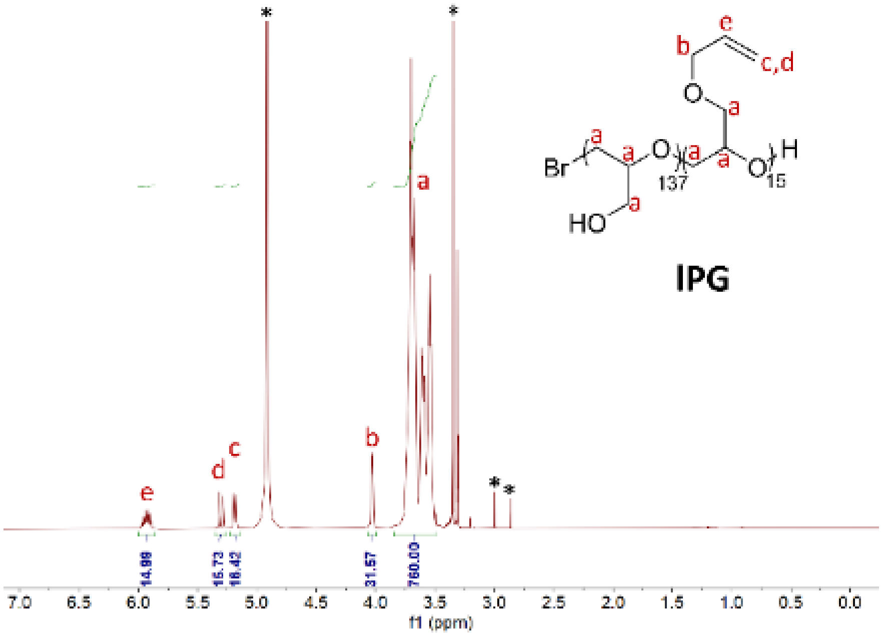

Figure S1. ^{1}H-NMR spectrum of **lPG** in CD_3OH (* denote peaks from residual solvents methanol, dimethylformamide and H_2O).

Synthesis of Sulfated PEEGE-PAGE (lPGS)

The hydroxyl groups on **lPG** was sulfated by following the reported procedure.[2] [1a] **lPG** (10 kDa, 0.84 g, 9.7 mmol OH groups, 1 eqv.) and SO_3-pyridine complex (4.6 g, 29 mmol, 3 eqv.)was dissolved in 120 mL DMF. The reaction mixture was stirred at 25 °C for 24 h. The mixture was condensed by evaporating DMF with a rotary evaporator at 45 °C under a vacuum of 10 mbar. Then 30 mL of deionized water was added to the condensed solution, and the pH was adjusted to 12 by adding saturated NaOH solution. The product was dialysed against brine and then deionized water. **lPGS** was obtained after lyophilization as a light-yellow powder. The polymer was characterized by ^{1}H NMR spectroscopy.

^{1}H-NMR (500 MHz, D_2O, 300 K) of **lPGS**: Repeating units δ = 3.57-4.00 (polymer backbone: C***H***$_2$-C***H***(CH$_2$O)-O, C***H***$_2$-C***H***-C***H***$_2$, O-C***H***2-CH=CH$_2$), 4.05-4.31 (CH$_2$-CH-C***H***$_2$-OSO$_3$Na), 5.20-5.44 (C***H***$_2$=CH-, 2H), 5.9-6.0 (CH2=C***H***-, 1H).

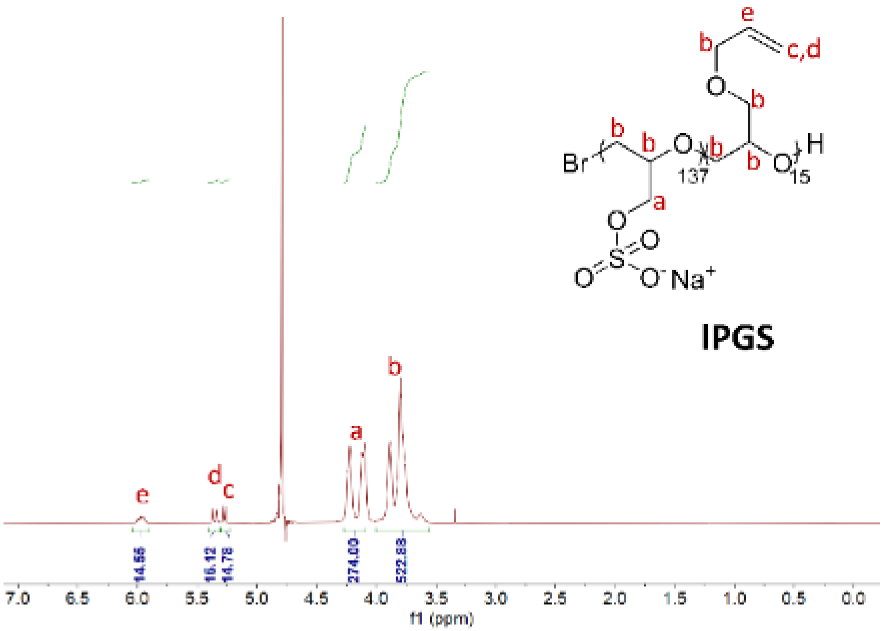

Figure S2. ^{1}H-NMR spectrum of **lPGS** in D_2O.

Synthesis of aminated lPGS (lPGS-NH_2)

Cysteamine was coupled with the allyl groups of **lPGS** via thiol-ene by UV irradiation (~25 mW/cm^2, λ= 370 nm) under ambient laboratory conditions to yield the amine groups on the polymer. **lPGS** (1.7 g, 1.28 mmol allyl group, 1 eqv.) and cysteamine hydrochloride (727 mg, 6.4 mmol, 4 eqv. to allyl groups) were dissolved into 65 mL deionized water. Then 2-Hydroxy-4′-(2-hydroxyethoxy)-2-methylpropiophenon (65 mg, Irgacure 259) was added and the reaction was conducted under the irradiation of 370nm UV light at room temperature. After 3 hours reaction, the product was purified by dialysis in deionized water. **lPGS-NH_2** was obtained after lyophilization as a light-yellow powder. The polymer was characterized by ^{1}H NMR spectroscopy.

^{1}H-NMR (500 MHz, D_2O, 300 K) of **lPGS-NH_2**: Repeating units δ = 4.04-4.31 (CH_2-CH-C$\boldsymbol{H}_2$-OSO_3Na, 2H), 3.57-3.98 (polymer backbone: C$\boldsymbol{H}_2$-C$\boldsymbol{H}$(CH_2O)-O, C$\boldsymbol{H}_2$-C$\boldsymbol{H}$-C$\boldsymbol{H}_2$, O-C$\boldsymbol{H}_2$-CH=CH_2), 3.2-3.3 (S-CH_2-C$\boldsymbol{H}_2$-N, 2H), 2.84-2.95 (S-C$\boldsymbol{H}_2$-CH_2-N, 2H), 2.63-2.77 (O-CH_2-CH_2-C$\boldsymbol{H}_2$-S, 2H), 1.82-1.97 (O-CH_2-C$\boldsymbol{H}_2$-CH_2-S, 2H).

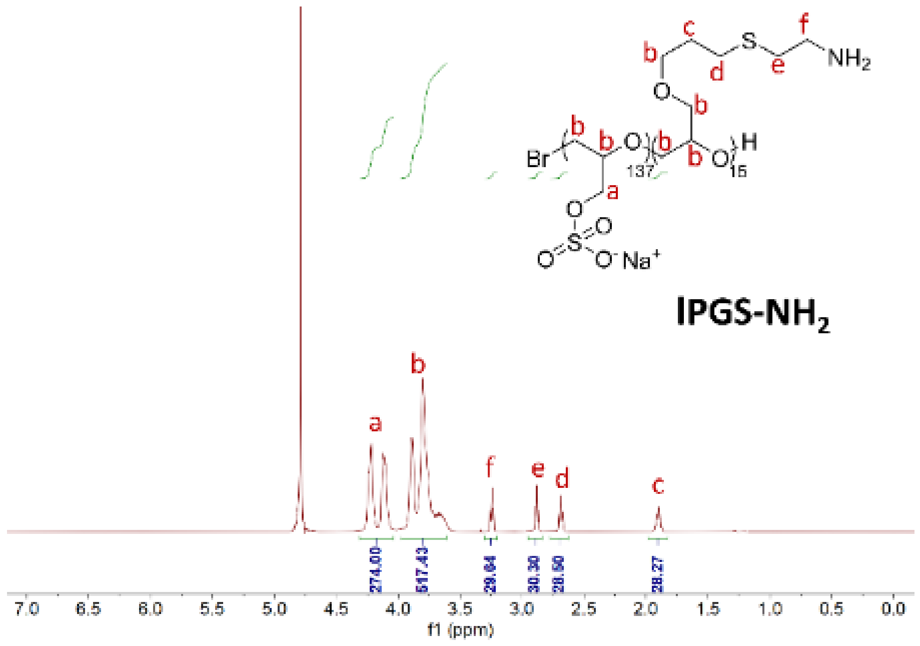

Figure S3. ^{1}H-NMR spectrum of **lPGS-NH$_2$** in D_2O.

Synthesis of lPGS-nTA

Next, the corresponding numbers of thioctic acid (**TA**) unit were coupled to **lPGS-NH$_2$** using NHS-ester chemistry. The thioctic acid NHS (**TA-NHS**) ester was synthesised according to the reported procedure.[3] The obtained **lPGS-nTA** were characterised by ^{1}H-NMR (Figure S1). The number of thioctic acid (**TA**) coupled to **lPGS-nTA** could be well controlled through tuning the equivalents of **TA-NHS** as this reaction step is quantitative.

To synthesize **lPGS-3TA**, **lPGS-NH$_2$** (1g, 20 KDa, 0.05 mmol) and **TA-NHS** (45.5 mg, 303 g/mol, 0.15 mmol) were dissolved with DMF (20 mL) and H_2O (4 mL) in a round flask. 200 μL of triethylamine as a catalyst was added to above system. The reaction mixture was stirred for 24 hrs at room temperature. The resulting mixture was then dialysed against light alkaline aqueous condition (pH 8, adjusted with NaOH) for 3 times and 2 times in deionized water. The resulting solution was lyophilised to get light yellow solid with 98-99% yield.

For **lPGS-5TA**, **lPGS-6TA** and **lPGS-15TA**, a similar synthetic procedure was applied. The only difference is for **lPGS-5TA**, **lPGS-6TA**, 5, 6 equivalents of **TA-NHS** was applied respectively, for **lPGS-15TA**, 15 equivalents of **TA-NHS** was applied in the reaction. All target polymers present as light-yellow solid. All the polymers were stored at -20 °C for use.

^{1}H-NMR (500 MHz, D_2O, 300 K) of **lPGS-3TA**: Repeating units δ = 4.01-4.42 (CH_2-CH-C***H***$_2$-OSO_3Na, 2H), 3.59-3.96 (polymer backbone: C***H***$_2$-C***H***(CH_2O)-O, C***H***$_2$-C***H***-C***H***$_2$, O-C***H***2-CH=CH_2; CH_2-C***H***-S-S, 1H), 3.39-3.43 (S-C***H***$_2$-CH_2-CH, 2H), 3.18-3.28 (S-CH_2-C***H***$_2$-N, 2H), 2.89-2.91 (S-C***H***$_2$-CH_2-N, 2H), 2.64-2.78 (O-CH_2-CH_2-C***H***$_2$-S, 2H), 2.47-2.55 (S-CH_2-C***H***$_2$-CH, 1H), 2.24-2.32 (O=C-C***H***$_2$-CH_2-, 2H), 1.98-2.05 (S-CH_2-C***H***$_2$-CH, 1H), 1.59-1.97 (O-CH_2-C***H***$_2$-CH_2-S, 2H; O=C-CH_2-C***H***$_2$-CH_2-CH_2-CH-S-S-, 2H; O=C-CH_2-CH_2-CH_2-C***H***$_2$-CH-S-S-, 2H), 1.41-1.49 (O=C-CH_2-CH_2-C***H***$_2$-CH_2-CH-S-S-, 2H).

^{1}H-NMR (500 MHz, D_2O, 300 K) of **lPGS-5TA**: Repeating units δ = 4.01-4.31 (CH_2-CH-C$\boldsymbol{H}_2$-OSO_3Na, 2H), 3.65-3.88 (polymer backbone: C$\boldsymbol{H}_2$-C***H***(CH_2O)-O, C$\boldsymbol{H}_2$-C***H***-C$\boldsymbol{H}_2$, O-C***H***2-CH=CH_2; CH_2-C***H***-S-S, 1H), 3.36-3.39 (S-C$\boldsymbol{H}_2$-CH_2-CH, 2H), 3.14-3.25 (S-CH_2-C$\boldsymbol{H}_2$-N, 2H), 2.82-2.88 (S-C$\boldsymbol{H}_2$-CH_2-N, 2H), 2.60-2.74 (O-CH_2-CH_2-C$\boldsymbol{H}_2$-S, 2H), 2.43-2.52 (S-CH_2-C$\boldsymbol{H}_2$-CH, 1H), 2.23-2.28 (O=C-C$\boldsymbol{H}_2$-CH_2-, 2H), 1.95-2.01 (S-CH_2-C$\boldsymbol{H}_2$-CH, 1H), 1.55-1.92 (O-CH_2-C$\boldsymbol{H}_2$-CH_2-S, 2H; O=C-CH_2-C$\boldsymbol{H}_2$-CH_2-CH_2-CH-S-S-, 2H; O=C-CH_2-CH_2-CH_2-C$\boldsymbol{H}_2$-CH-S-S-, 2H), 1.39-1.46 (O=C-CH_2-CH_2-C$\boldsymbol{H}_2$-CH_2-CH-S-S-, 2H).

^{1}H-NMR (500 MHz, D_2O, 300 K) of **lPGS-6TA**: Repeating units δ = 4.01-4.37 (CH_2-CH-C$\boldsymbol{H}_2$-OSO_3Na, 2H), 3.57-3.94 (polymer backbone: C$\boldsymbol{H}_2$-C***H***(CH_2O)-O, C$\boldsymbol{H}_2$-C***H***-C$\boldsymbol{H}_2$, O-C***H***2-CH=CH_2; CH_2-C***H***-S-S, 1H), 3.38-3.44 (S-C$\boldsymbol{H}_2$-CH_2-CH, 2H), 3.16-3.29 (S-CH_2-C$\boldsymbol{H}_2$-N, 2H), 2.86-2.92 (S-C$\boldsymbol{H}_2$-CH_2-N, 2H), 2.63-2.76 (O-CH_2-CH_2-C$\boldsymbol{H}_2$-S, 2H), 2.47-2.54 (S-CH_2-C$\boldsymbol{H}_2$-CH, 1H), 2.24-2.32 (O=C-C$\boldsymbol{H}_2$-CH_2-, 2H), 1.98-2.05 (S-CH_2-C$\boldsymbol{H}_2$-CH, 1H), 1.58-1.97 (O-CH_2-C$\boldsymbol{H}_2$-CH_2-S, 2H; O=C-CH_2-C$\boldsymbol{H}_2$-CH_2-CH_2-CH-S-S-, 2H; O=C-CH_2-CH_2-CH_2-C$\boldsymbol{H}_2$-CH-S-S-, 2H), 1.40-1.51 (O=C-CH_2-CH_2-C$\boldsymbol{H}_2$-CH_2-CH-S-S-, 2H).

^{1}H-NMR (500 MHz, D_2O, 300 K) of **lPGS-15TA**: Repeating units δ = 4.01-4.42 (CH_2-CH-C$\boldsymbol{H}_2$-OSO_3Na, 2H), 3.59-3.94 (polymer backbone: C$\boldsymbol{H}_2$-C***H***(CH_2O)-O, C$\boldsymbol{H}_2$-C***H***-C$\boldsymbol{H}_2$, O-C***H***2-CH=CH_2; CH_2-C***H***-S-S, 1H), 3.38-3.44 (S-C$\boldsymbol{H}_2$-CH_2-CH, 2H), 3.17-3.30 (S-CH_2-C$\boldsymbol{H}_2$-N, 2H), 2.71-2.77 (S-C$\boldsymbol{H}_2$-CH_2-N, 2H), 2.63-2.70 (O-CH_2-CH_2-C$\boldsymbol{H}_2$-S, 2H), 2.47-2.56 (S-CH_2-C$\boldsymbol{H}_2$-CH, 1H), 2.24-2.32 (O=C-C$\boldsymbol{H}_2$-CH_2-, 2H), 1.98-2.05 (S-CH_2-C$\boldsymbol{H}_2$-CH, 1H), 1.58-1.94 (O-CH_2-C$\boldsymbol{H}_2$-CH_2-S, 2H; O=C-CH_2-C$\boldsymbol{H}_2$-CH_2-CH_2-CH-S-S-, 2H; O=C-CH_2-CH_2-CH_2-C$\boldsymbol{H}_2$-CH-S-S-, 2H), 1.40-1.50 (O=C-CH_2-CH_2-C$\boldsymbol{H}_2$-CH_2-CH-S-S-, 2H).

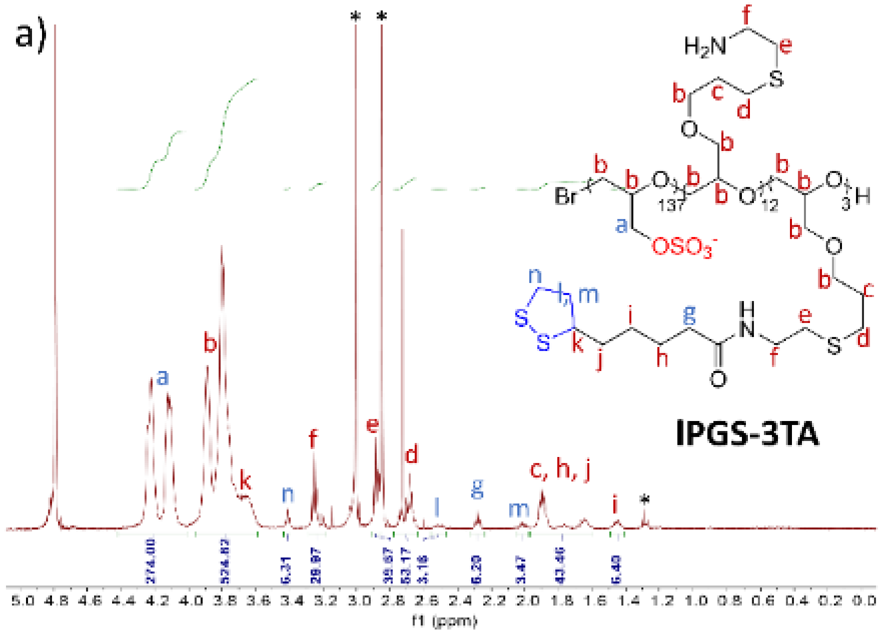

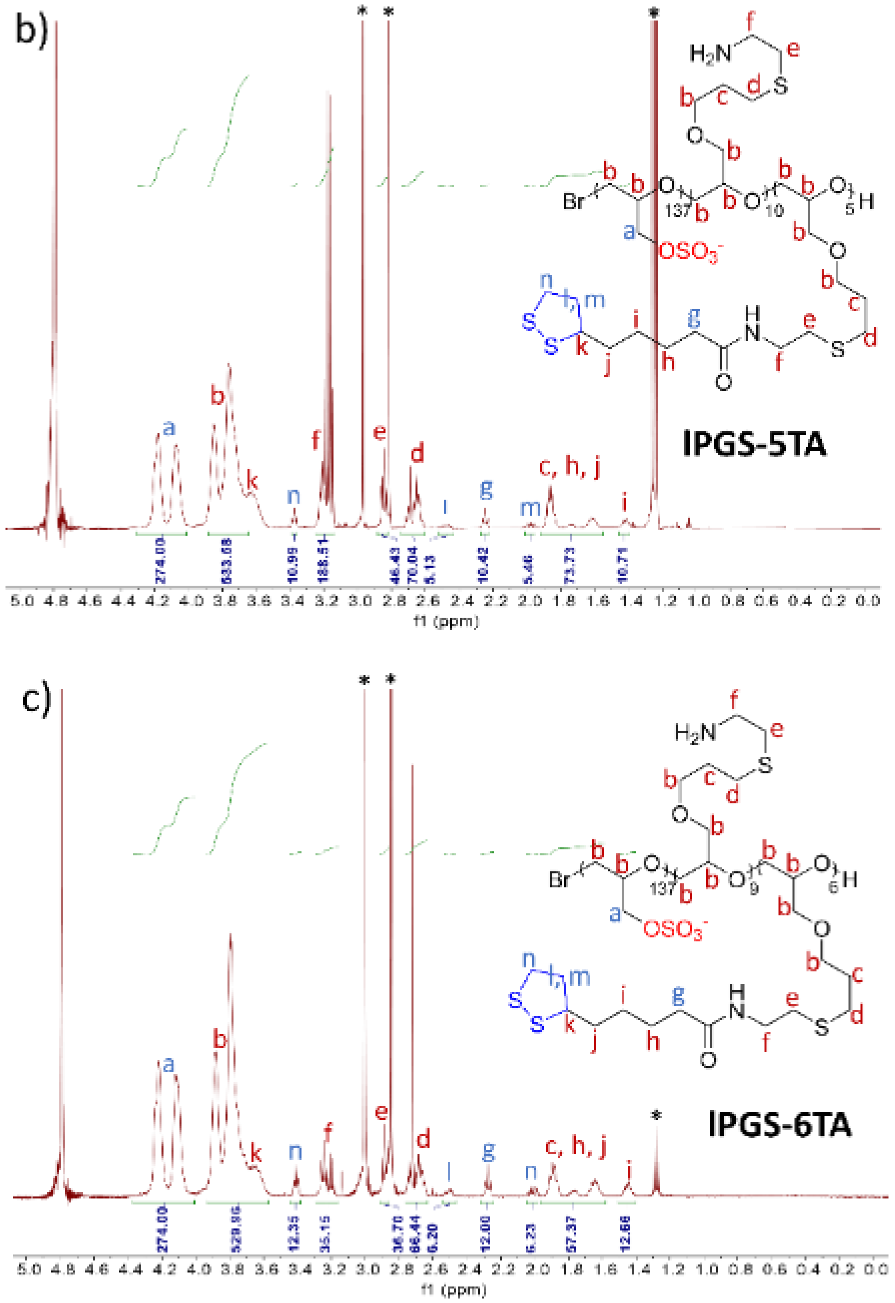
b)
IPGS-5TA
H2N
OSO3-
Br
137
10
5
H
a
b
c, h, j
d
e
f
g
i
k
l
m
n
274.00
533.68
10.99
188.51
46.43
70.04
5.13
10.42
5.46
73.73
10.71
f1 (ppm)
c)
IPGS-6TA
137
9
6
274.00
529.86
12.35
35.15
36.70
66.44
6.20
12.00
6.23
57.37
12.66
f1 (ppm)

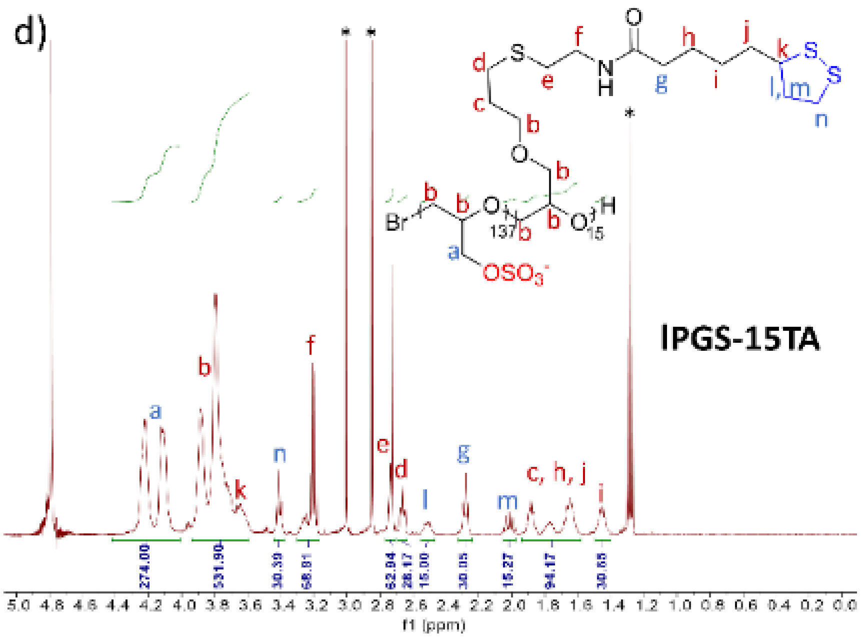

Figure S4. ^{1}H-NMR spectra of **lPGS-nTA (n = 3, 5, 6, 15)** in D_2O. * denote peaks from residual solvents dimethylformamide and H_2O. In the spectra, the integrals of certain peaks, labelled with blue letters (a, n, l, g, m), can be clearly assigned. However, the integrals of other protons, especially those from the alkyl chain, do not exactly match the actual number of protons due to overlap with peaks from solvent residues or polymer backbone. The integral of protons from -C**H**$_2$- group adjacent to the -OSO_3- on the backbone (a) was used as a reference. The NMR spectra indicate that thiotic acid (**TA**) was quantitatively coupled to **lPGS-NH$_2$**, as evidenced by the integrals of characteristic protons (n, l, g, m) from thiotic acid (**TA**).

Time-resolved NMR

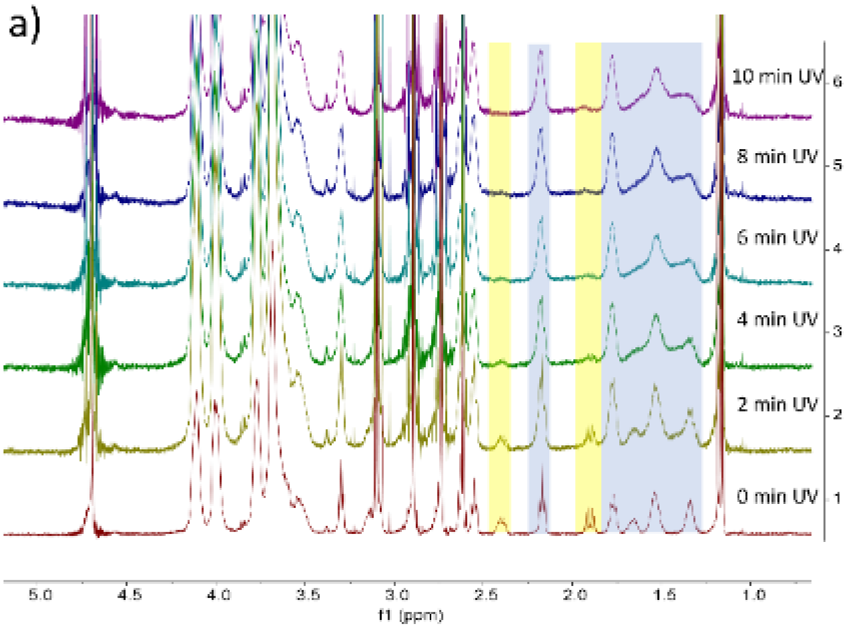

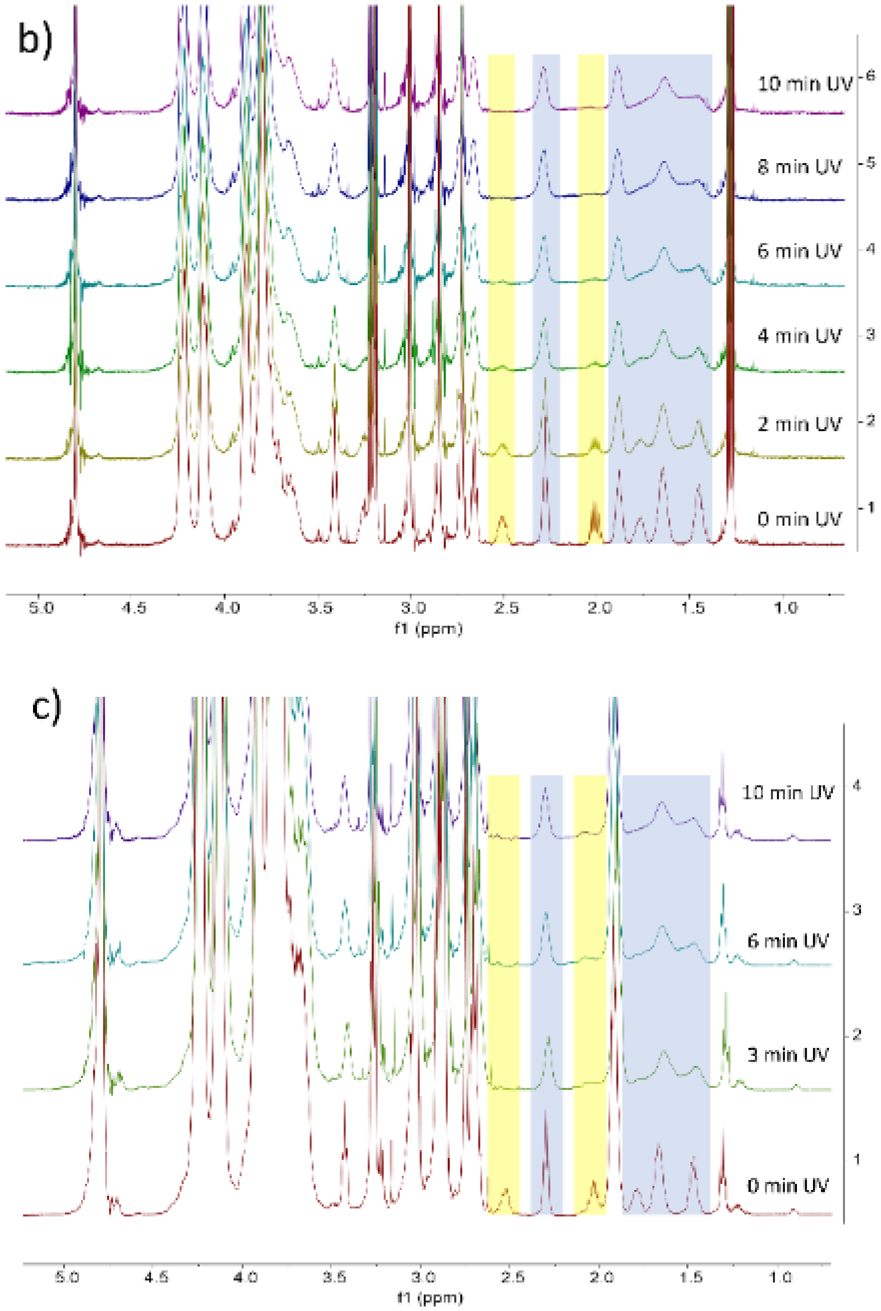

Figure S5. a) time-resolved NMR of **lPGS-15TA** under UV exposure at 3% (w/v). b) time-resolved NMR of **lPGS-15TA** under UV exposure at 6% (w/v). c) time-resolved NMR of **lPGS-3TA** under UV exposure at 10% (w/v). Peaks labelled by yellow colour are the characteristic peaks (as shown in Figure S1c protons b/c) from the five-member ring of **TA**. Upon UV irradiation the five-member ring opens and crosslink intermolecularly, which leads to the decrease till disappear of the integration. Meanwhile, the intermolecular disulfide crosslinking also leads to the merge of other well split peaks from the alkyl chain of **TA** as labelled by blue colour.

Gel preparation and Summary of gelation behaviour of lPGS-nTA

The light-responsive polymers (**lPGS-nTA)** were dissolved in PBS buffer (pH 7.4, 10 mM) at the corresponding weight concentration in a 2 mL glass vial. In the next step, solutions were exposed to 370 nm UV light (25 mW/cm^2) for gelation and status of the vials were evaluated visually.

Table S1. Gelation study of **lPGS-nTA** at different concentration and different UV exposure time.

Compound	Number of **TA** units	Concentration	UV exposure time	State	
				Before UV	After UV
lPGS-3TA	3	10% w/v	60 min	Solution	Solution
lPGS-5TA	5	10% w/v	10 min	Solution	Free-standing gel
lPGS-6TA	6	8% w/v	5 min	Solution	Free-standing gel
LPGS-15TA	15	5% w/v	5 min	Solution	Free-standing gel

Oscillatory Rheology of lPGS-nTA hydrogels

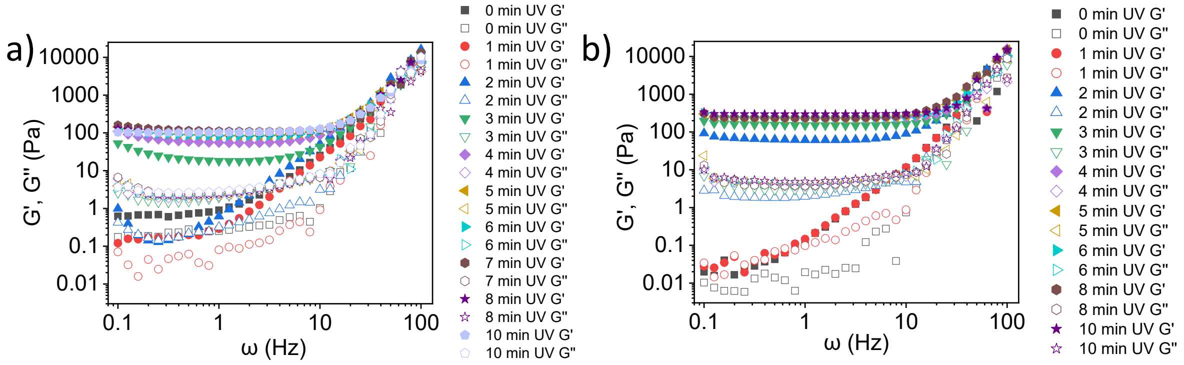

Figure S6. Storage (G´) and loss (G´´) moduli as function of radial frequency ω for **lPGS-nTA** after different UV light exposure time. a) **lPGS-6TA** (8% w/v) and b) **lPGS-15TA** (5% w/v). All the experiments were conducted triplet. The error bars were omitted for clarity.

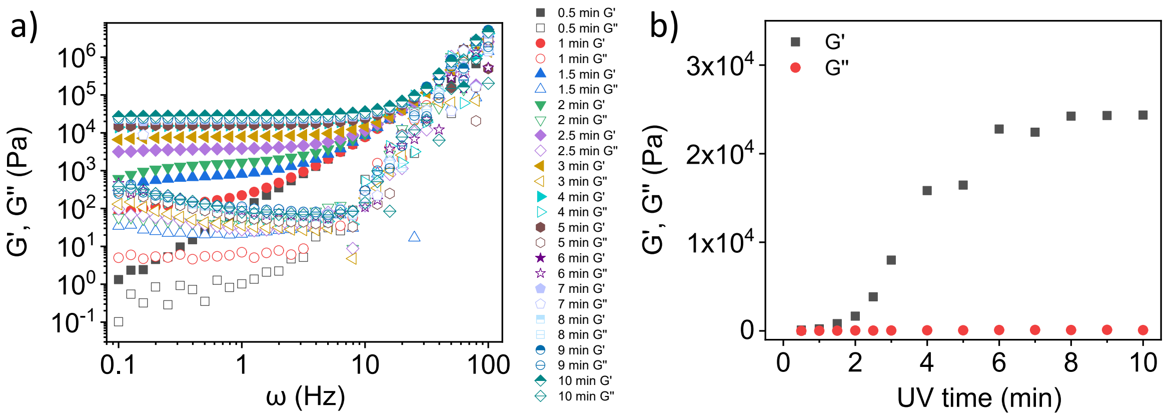

Figure S7. a) Storage (G´) and loss (G´´) moduli as function of radial frequency ω for **lPGS-15TA** (10% w/v) after different UV light exposure time. b) Change in the storage moduli (G´) and loss moduli (G´´) at 1 Hz as a function of UV light exposure time for **lPGS-15TA** (10% w/v).

Scanning Electron Microscope (SEM) images of lPGS-nTA

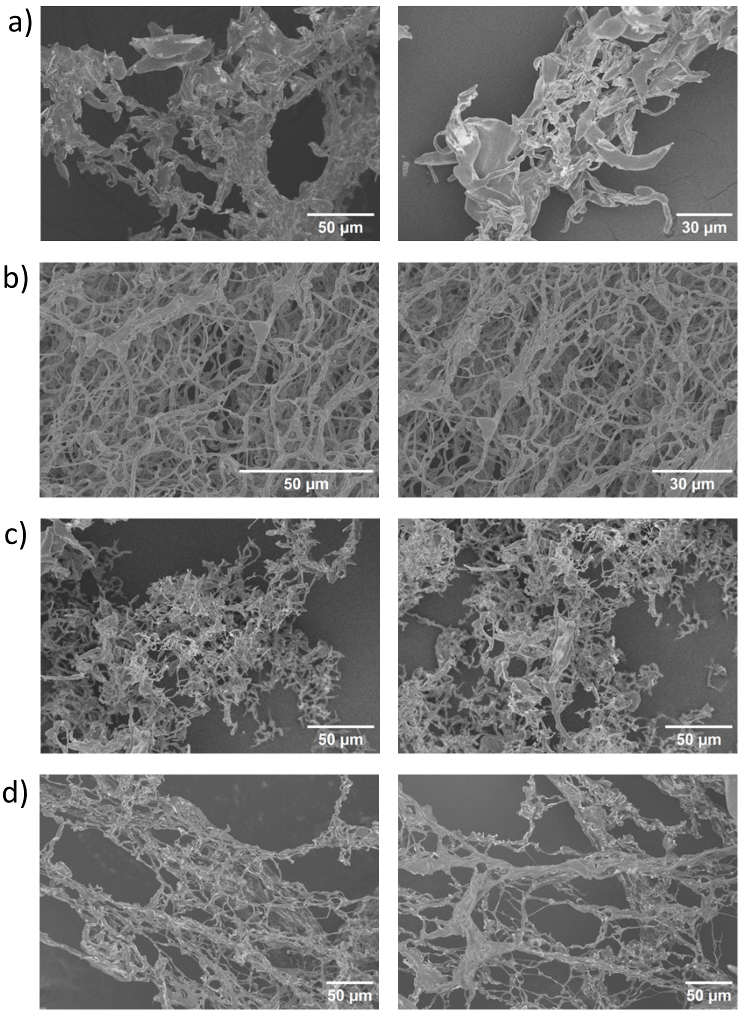

Figure S8. More SEM images of **lPGS-15TA** (5% w/v) and **lPGS-6TA** (8% w/v). a) **lPGS-15TA** (5% w/v) before UV irradiation. b) **lPGS-15TA** (5% w/v) after 5 min UV irradiation. c) **lPGS-6TA** (8% w/v) before UV irradiation. d) **lPGS-6TA** (8% w/v) after 5 min UV irradiation.

Oscillatory Rheology of mixture of lPGS-15TA and BSM

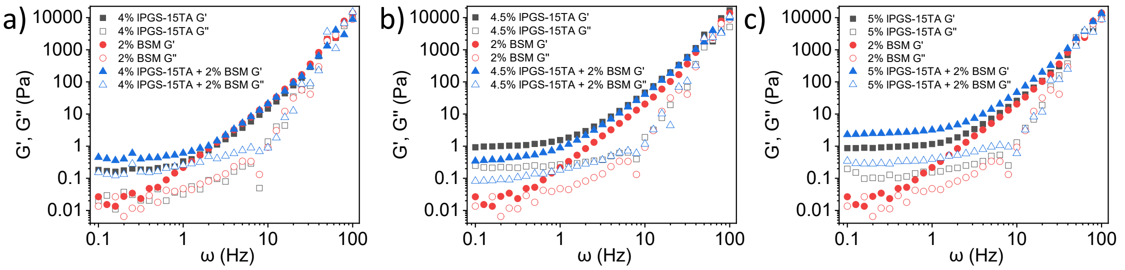

Figure S9. Storage (G´) and loss (G´´) moduli as function of radial frequency ω for **lPGS-15TA** at different concentrations, **BSM** (2%) and their mixture before irradiation. All the experiments were conducted in triplicate. The error bars were omitted for clarity.

Scanning Electron Microscope (SEM) images of biohybrid hydrogels

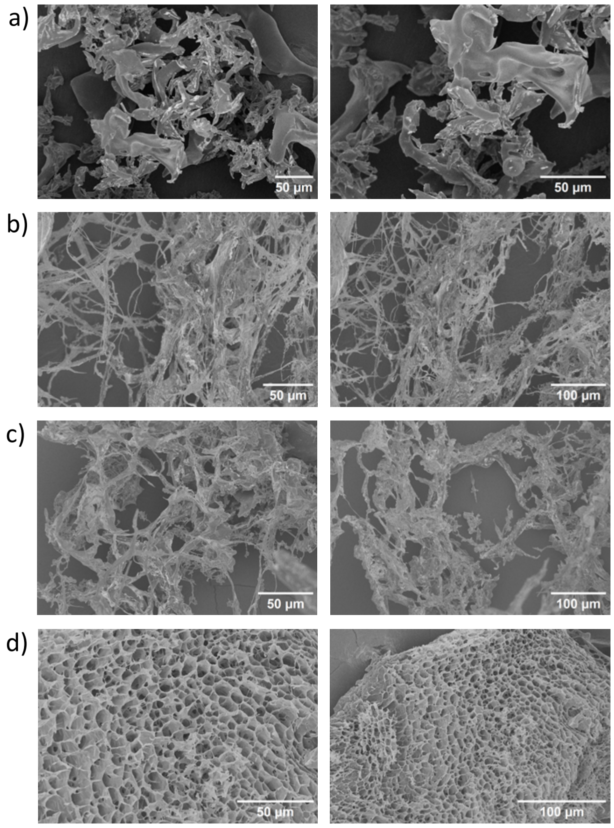

Figure S10. More SEM images of **IPGS-15TA** (4% w/v) and **BSM** (2% w/v) and the mixture. a) **IPGS-15TA** (4% w/v) before UV irradiation. b) **BSM** (2% w/v) without UV irradiation. c) the mixture of **IPGS-15TA** (4% w/v) and **BSM** (2% w/v) without UV irradiation. d) the mixture of **IPGS-15TA** (4% w/v) and **BSM** (2% w/v) after 5 min UV irradiation.

Oscillatory Rheology of biohybrid hydrogels

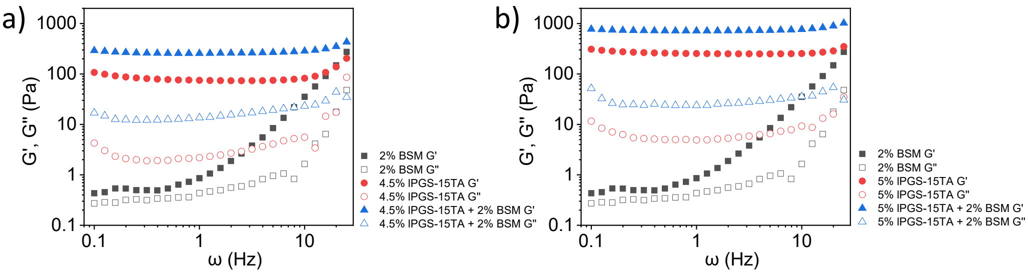

Figure S11. Storage (G´) and loss (G´´) moduli as function of radial frequency ω for **IPGS-15TA** at different concentrations, **BSM** (2%) and their mixture upon 5 min UV irradiation. All the experiments were conducted in triplicate. The error bars were omitted for clarity.

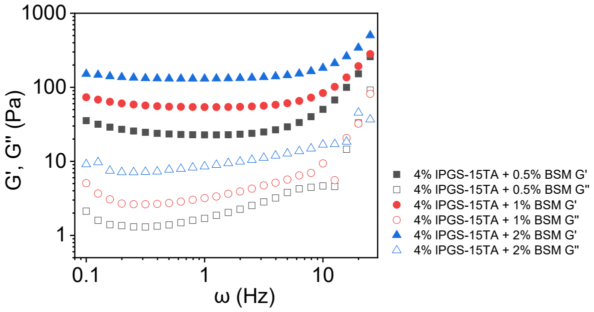

Figure S12. Storage (G´) and loss (G´´) moduli as function of radial frequency ω for mixture of **IPGS-15TA** (4%) with different concentrations of **BSM** upon 5 min UV irradiation. All the experiments were conducted in triplicate. The error bars were omitted for clarity.

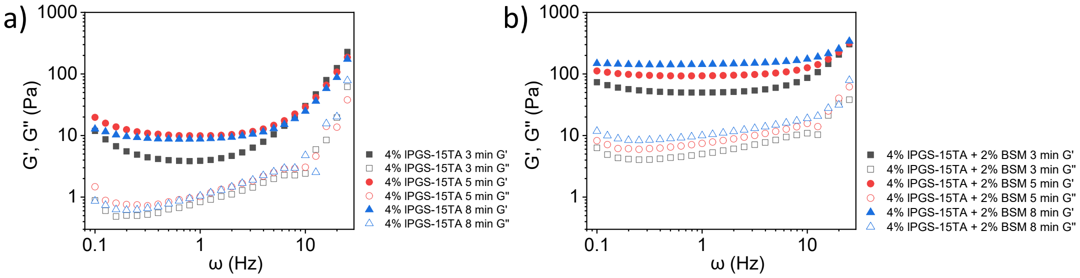

Figure S13. Storage (G´) and loss (G´´) moduli as function of radial frequency ω for a) **IPGS-15TA** (4%) upon different time of UV irradiation, and b) a mixture of **IPGS-15TA** (4%) and **BSM** (2%) upon different time of UV irradiation. All the experiments were conducted in triplicate. The error bars were omitted for clarity.

References

[1] a) J. Feng, C. Nie, E. Xie, B. Thongrom, V. Reiter-Scherer, S. Block, A. Herrmann, E. Quaas, C. Sieben, R. Haag, *ACS Appl. Mater. Interfaces.* **2023**, *15*, 51894-51904; b) L. Yu, C. Cheng, Q. Ran, C. Schlaich, P.-L. M. Noeske, W. Li, Q. Wei, R. Haag, *ACS Appl. Mater. Interfaces.* **2017**, *9*, 6624-6633.

[2] H. Türk, R. Haag, S. Alban, *Bioconjug. Chem.* **2004**, *15*, 162-167.

[3] B. Sieredzinska, Q. Zhang, K. J. v. d. Berg, J. Flapper, B. L. Feringa, *Chem. Commun.* **2021**, *57*, 9838-9841.
